# The cardiac response of the goldfish *Carassius auratus* to environmental hypoxia: from hemodynamics to mitochondria

**DOI:** 10.1007/s10695-025-01452-8

**Published:** 2025-01-24

**Authors:** Mariacristina Filice, Rosa Mazza, Alfonsina Gattuso, Alessia Caferro, Gaetana Napolitano, Gianluca Fasciolo, Paola Venditti, Sandra Imbrogno, Maria Carmela Cerra

**Affiliations:** 1 Department of Biology, Ecology and Earth Science, University of Calabria, Rende, Italy; 2 Department of Science and Technology, Parthenope University of Naples, Naples, Italy; 3 Department of Biology, University of Naples Federico II, Naples, Italy

**Keywords:** Fish heart, Hypoxia, Mitochondrial biogenesis, Oxygen consumption

## Abstract

Under low O_2_, the heart of *Carassius auratus* (goldfish) shows an enhanced hemodynamics. This is observed in ex vivo cardiac preparations from animals acclimated to both normoxia and short-term (4 days) moderate hypoxia and perfused for 90 min with a hypoxic medium. Under short-term hypoxia, this is associated with a higher ventricular muscularity and an expanded mitochondrial compartment. To date, little is known about the putative influence of hypoxia on the mitochondrial contribution to cardiac energy metabolism. Similarly, it remains unexplored whether the exposure to environmental low O_2_ affects the cardiac response to preload increases (i.e., the Frank–Starling mechanism). We here observed, on ex vivo isolated and perfused goldfish heart, that 20 days of exposure to moderate water hypoxia are accompanied by a potentiated cardiac performance, analyzed as stroke volume, cardiac output, and stroke work. The sensitivity to preload increases significantly improved after 20 days of hypoxia, while it is similar to normoxia after 4 days of exposure. This suggested a time-dependent response. Mitochondrial O_2_ consumption initially decreased during short-term hypoxia but returned to normoxia-like levels after 20 days of exposure. Biomolecular analyses of ventricular extracts revealed a time-dependent regulation of key proteins involved in the mitochondrial biogenesis, including PGC1α, NRF1/2, and TFAM, as well as cytochrome c. Additionally, mitochondrial DNA content was notably increased after 20 days of hypoxia. Our data revealed that, when challenged by chronic environmental hypoxia, the goldfish heart improves its pumping behavior under both basal and loading-stimulated conditions. This is accompanied by a mitochondrial remodeling which likely supports adequate energy supply for the working myocardium.

## Introduction

The ability to tolerate water O_2_ limitations is an adaptive trait of several teleost species. It represents an evolutionary advantage since it allows fish to colonize environments with poor O_2_ availability. Among tolerant fish, members of the cyprinid genus Carassius, such as the crucian carp (*Carassius carassius*) and the goldfish (*Carassius auratus*), exhibit an impressive natural ability to remain active and survive for long periods in the presence of reduced O_2_, even up to complete anoxia (Bickler and Buck 2007). This is allowed by several functional mechanisms that include metabolic depression of approximately 30% (Shoubridge and Hochachka 1980), glucose mobilization from hepatic glycogen stores, ethanol production by anaerobic metabolism, and acidosis avoidance through ethanol removal via the branchial epithelium (Cerra et al. 2023; Filice et al. 2022; Gattuso et al. 2018). A key aspect of the cyprinids hypoxia tolerance, well-documented in both the crucian carp and the goldfish, is the remarkable functional and metabolic plasticity of the heart that allows the fish to satisfy peripheral energy requirements, also supporting the integration between tissues and organs (Imbrogno et al. 2014; Stecyk et al. 2004). In particular, the goldfish heart perfused under acute hypoxia (2.5 ± 0.3 mg O_2_ l^−1^) is characterized by an enhancement of the basal performance, revealed by a time-dependent increase of stroke volume (*V*_S_) (Imbrogno et al. 2014). Additionally, an improved hemodynamic behavior is evident when the perfused goldfish heart is exposed to preload increments (i.e., the Frank–Starling response). Under these conditions, the maximum *Vs* value is achieved at an input pressure lower than the normoxic counterparts (Imbrogno et al. 2014). Of interest, recent findings from our laboratory (Filice et al. 2024b) demonstrated that goldfish exposed to a short period (4 days) of water hypoxia (PO_2_ of about 34 mmHg) exhibit an improved cardiac hemodynamics, revealed by basal *V*_S_ values higher than those detected in normoxic fish. These functional changes are accompanied by morphological remodeling of the ventricular pump which, in the heart from hypoxia-exposed animals, is more muscular and vascularized; thus, it is better suited to sustain the potentiated performance. Interestingly, the ventricular myocardiocytes of hypoxia-exposed goldfish show a remodeling of the energy apparatus, represented by an increased number of mitochondria and a modulation of proteins controlling mitochondrial fission (Filice et al. 2024b). An involvement of mitochondria dynamics in the intracellular mechanisms activated by hypoxia has been also observed in the heart of goldfish exposed to prolonged severe hypoxia (4 weeks, PO₂ ≈ 15 mmHg); however, in this case, an increment of transcripts related to pro-fusion proteins has been reported (Farhat et al. 2022). Thus, it is conceivable that the degree of O_2_ deprivation and/or the duration of exposure may influence the mitochondrial response of goldfish myocardiocytes to hypoxia. This is of relevance since a time-dependent modulation of mitochondrial dynamics (fission vs. fusion vs. biogenesis), by regulating mitochondria number and mtDNA distribution, is crucial to maintain cell respiratory activity when O_2_ availability becomes a limiting factor.

Based on these premises, the present study was designed to investigate the functional and molecular responses of the goldfish heart to 20 days of moderate water hypoxia (PO_2_ ~ 34 mmHg). Together with our previous findings (Filice et al. 2024b; Imbrogno et al. 2014), results may contribute to a better understanding of the mechanisms that allow the goldfish heart to face hypoxia and are also useful to provide a time-dependent framework. This is particularly relevant to current scientific research, given the growing use of this cyprinid as a model organism in both translational and eco-physiological studies. This study offers a valuable opportunity to decipher the network of events that, unlike the more vulnerable mammalian myocardium, empowers the goldfish heart to withstand O_2_ deprivation (Filice et al. 2022). At the same time, our results may also shed light on whether and to what extent the fish heart is challenged by the reduction in water O_2_ due to the increasing environmental deterioration.

To this aim, goldfish were exposed for 20 days to PO_2_ values above their critical oxygen tension (P*crit*) [30 mmHg (Fry and Hart 1948; Hansen and Jensen 2010)], and the basal cardiac performance was evaluated. In addition, animals exposed for 4 and 20 days to the same PO_2_ were used to analyze (i) the response of the heart to increased preloads (i.e., Frank–Starling response), (ii) the ability of cardiac mitochondria to use O_2_, (iii) the expression of markers involved in mitochondrial biogenesis (i.e., cytochrome c, PGC1α-NRF1/2-TFAM), and (iv) the levels of reactive oxygen species (ROS).

## Materials and methods

### Animals

Goldfish (*C. auratus*; length = 10–12 cm; weight = 36.88 ± 12.31 g; means ± s.d.; *N* = 85) of both sexes were provided by a local fish farm (CARMAR, Italy). They were maintained under a 12-h light/dark cycle at 21–22 °C in filtered, aerated, and dechlorinated tap water, and daily fed with commercial food (Premium Gold, Vitakraft, Germany). Animal care and experimental procedures were in accordance with European and Italian laws and approved by the Institutional Animal Care and Use Committee (CESA) of the University of Naples Federico II, Naples, Italy (N. 767/2023-PR).

### Normoxia/hypoxia exposure

Animals were randomly assigned to one of the following three groups: normoxia (*N* = 32), short hypoxia (Hypo_4d; *N* = 21), or chronic hypoxia (Hypo_20d; *N* = 32). The normoxic group was maintained at a PO_2_ of about 150 mmHg, while the hypoxic groups were kept at PO_2_ values of 34 ± 3 mmHg, above the critical goldfish O_2_ tension [30 mmHg: (Fry and Hart 1948; Hansen and Jensen 2010)]. PO_2_ values were obtained by bubbling air (normoxia) or N_2_ (hypoxia) into the water. In the hypoxic tanks, PO_2_ decreased to the desired level within 5–6 h and then maintained stable at values of 34 ± 3 mmHg. To minimize gas exchange between water and air, aquaria were filled almost to the brim and covered with a plexiglass lid. Water flow was set to 10 ml min^−1^, and O_2_ levels were recorded several times a day using an O_2_ analyzer (Milwaukee, SM600, Szeged, Hungary) [see also (Filice et al. 2024b)]. The water temperature was kept at 21–22 °C.

At the end of the exposure period, animals were anesthetized with MS222 (tricaine methanesulfonate; 0.2 g l^−1^) (Sigma Aldrich, Milan, Italy) and sacrificed. They were weighed (normoxia: 38.3 ± 2.0 g; Hypo_4d: 37.0 ± 3.0; Hypo_20d: 35.3 ± 2.3) and then ventrally opened behind the pectoral fins. The heart was excised and properly processed.

### Ex vivo working heart preparation

Hearts were excised without the pericardium and immediately placed in a saline-filled dish for the cannulation procedure. Two polyethylene *cannulae* were inserted into the *sinus venosus*, for the inflow, and into the ventral aorta, for the outflow collection, respectively. The cannulated heart was then transferred into a perfusion chamber filled with saline and connected to a perfusion apparatus. The saline composition was (in mmol l^−1^) NaCl 124.9, KCl 2.49, MgSO_4_ 0.94, NaH_2_PO_4_ 1.0, glucose 5.0, NaHCO_3_ 15.0, and CaCl_2_ 1.2 (Garofalo et al. 2012). pH was adjusted to 7.7–7.9. The heart received saline from an input reservoir and pumped it against an afterload pressure given by the height of an output reservoir.

Hearts from normoxic and hypoxic animals were perfused with normoxic or hypoxic saline, respectively. To this end, the PO_2_ of the saline in the main reservoir was maintained at a value of ≈ 150 mmHg (normoxia) or ≈ 30 mmHg (hypoxia) to ensure a PO_2_ reaching the heart of ≈ 100 mmHg, or ≈ 25 mmHg, respectively (Filice et al. 2022). O_2_ concentration was continuously monitored by an O_2_ analyzer (Milwaukee, SM600, Szeged, Hungary). All experiments were conducted at 20–21 °C.

The isolated and perfused heart was allowed to generate its own rhythm. The mean output pressure was set at 1.5 kPa, and the filling pressure was adjusted to about 0.07 kPa (Filice et al. 2020). Pressures were measured by using an MP-20D pressure transducer (Micron Instruments, Simi Valley, CA, USA), connected to a PowerLab data acquisition system; they were analyzed using the Chart software (ADInstruments Basile, Comerio, Italy) and corrected for cannula resistance. Cardiac output (*Q̇*) was collected over 1 min and weighed. Values were corrected for fluid density and expressed as volume measurements normalized for body weight (ml min^−1^ kg^−1^). Heart rate (*f*_H_, beats min^−1^) was obtained from digital pressure traces. Stroke volume (*V*_S_; ml kg^−1^), the ratio between *Q̇* and *f*_H_, was used as a measure of ventricular performance; ventricular stroke work [*W*_S_; mJ g^−1^; (afterload–preload) *V*_S_ ventricle mass^−1^] served as an index of systolic functionality.

### Hemodynamic study

#### Basal performance

To assess the endurance of cardiac preparations, hearts from animals acclimated to either normoxia or 20 days of hypoxia were perfused under basal conditions; cardiac performance variables were measured at 10-min intervals over 90 min of perfusion period.

#### Frank–Starling response

Hearts from each experimental group (normoxia, Hypo_4d, and Hypo_20d) were used to study the response to preload increases. After stabilization, preload was incrementally increased by 0.5 cmH₂O until no further discernible increases in *Q̇* were observed. Cardiac performance variables were recorded after 5 min of perfusion at each preload increment. The output pressure was stable at 1.5 kPa.

### In vitro O_2_ consumption and cytochrome oxidase (COX) activity

The analysis of the oxidative metabolism was carried out on homogenates of cardiac tissue. Briefly, after animal sacrifice (*N* = 12 goldfish for each condition), the heart was rapidly removed and placed in an ice-cold isolation medium (IM) (220 mM mannitol, 70 mM sucrose, 1 mM EDTA, 20 mM Tris, pH 7.4) containing 0.1% fatty acid-free albumin. The heart from three goldfish was weighed, pooled, finely minced, and washed with IM. Tissue fragments were gently homogenized in IM (1:10 w/v) using a Potter–Elvehjem homogenizer set at a standard velocity (500 rpm) for 2 min.

O_2_ consumption was monitored on heart muscle homogenates in the presence (State 3) and the absence (State 4) of 750 μM ADP at 22 °C by a Hansatech respirometer in 0.5 ml of incubation medium (145 mM KCl, 30 mM Hepes, 5 mM KH_2_PO_4_, 3 mM MgCl_2_, 0.1 mM EGTA, 0,1% BSA, pH 7.4), using 0.0025 g of tissue homogenate and pyruvate/malate (10/2.5 mM) as substrates. State 4 O_2_ consumption was obtained in the presence of 2 μg/ml of oligomycin. O_2_ consumption was expressed as µmol O min^−1^ g^−1^ tissue.

Complex IV (cytochrome c oxidase) activity (COX) was determined by a polarographic procedure according to Napolitano et al. (2022). In brief, to 1.0 ml of incubation medium (145 mM KCl, 30 mM Hepes, 5 mM KH_2_PO_4_, 3 mM MgCl_2_, 0.1 mM EGTA, pH 7.4), 0.1 mg of homogenate tissue, solubilized with 1% Lubrol, was added. After stabilization, the reaction was started by adding a mixture of TMPD plus Ascorbate (30 mM plus 400 mM, respectively). COX activity was measured at 22 °C and expressed as μmol O min^−1^ mg^−1^ protein. The respiratory control ratio (RCR) was calculated as the ratio of State 3 to State 4 O_2_ consumption.

### ROS content and lipid oxidative damage

ROS content was evaluated by measuring, with a multimode microplate reader (Synergy™ HTX Multimode Microplate Reader, BioTek, λ_Ex_ 485, λ_Em_ 530 nm), the conversion induced by ROS of 2′,7′-dichlorodihydrofluorescin diacetate (DCFH-DA) in dichlorofluorescein (DCF) at 22 °C, as previously described (Napolitano et al. 2022). In brief, 12.5 µg of homogenate proteins were added to 200 μl of monobasic phosphate buffer 0.1 M, pH 7.4, and incubated for 15 min with 10 µM DCFH-DA. After the incubation period, 100 µM FeCl_3_ was added to the mixture and incubated for 30 min. ROS content was expressed as RFU (Relative Fluorescence Unit) mg^−1^ protein.

The extent of lipid peroxidation was assessed by determining lipid hydroperoxide (Hps) levels in cardiac homogenates (Heath and Tappel 1976). The reduction in NADPH absorbance due to reactions catalyzed by the enzymes glutathione peroxidase and glutathione reductase was followed at 340 nm in the presence of GSH. Measures were performed using a multimode microplate reader (Synergy™ HTX Multimode Microplate Reader, BioTek). Hps levels were reported as nmol NADPH min^−1^ gr tissue.

### Western blotting and densitometric analysis

Isolated hearts were homogenized in an ice-cold homogenization buffer (250 mM sucrose, 30 mM Tris, 1 mM EDTA, 1% SDS, pH 7.4), supplemented with Tissue Protease Inhibitor Cocktail (1:500 v/v, Sigma-Aldrich, Milan, Italy), 20 mM phenylmethylsulfonyl fluoride, and 200 mM sodium ortho-vanadate. The homogenates were then centrifuged at 10,000 × *g* for 10 min at 4 °C to remove tissue debris. Protein concentration in the supernatant was determined using the Bradford reagent (Sigma–Aldrich, Milan, Italy), according to the manufacturer. Western blotting was performed as previously described (Leo et al. 2019). Briefly, an amount of 60 μg protein for each homogenate was separated on polyacrylamide gels and electroblotted onto a PVDF membrane (GE Healthcare, Milan, Italy). For immunodetection, blots were incubated overnight at 4 °C with the following commercially available antibodies (Santa Cruz Biotechnology Inc., Texas, USA, or Novus Biologicals, Centennial, CO, USA): PGC1⍺ (cat# SC-13067), NRF1 (cat# SC-33771), NRF2 (cat# SC-22810), TFAM (cat# SC-28200), CytC (cat# SCSC-7159), and Drp1 (cat# NB110-55288). β-Actin (cat# SC-69879; dil. 1:2000) antibody was used as loading control. Immunodetection was performed by using peroxidase-linked secondary antibodies and an enhanced chemiluminescence kit (ECL PLUS, GE Healthcare, Milan, Italy). Quantitative band densitometry was performed on ChemiDoc images or digital images of X-ray films exposed to immunostained membranes; signal quantification was performed by Un-Scan-It gel software (Silk Scientific, Version 4.1, Provo, UT, USA). Results were expressed as means ± s.e.m. of absolute values.

### Determination of mtDNA/nDNA ratio

For both normoxia and Hypo_20d group, the mtDNA content, expressed as the mitochondrial to nuclear genome ratio (mtDNA/nDNA), was quantified using quantitative Real-Time PCR as described by Quiros et al. (2017) and detailed in Filice et al. (2024b).

Primers used for quantitative Real-Time PCR are listed in Table 1. Amplification reactions were performed via SYBR™ Select Master Mix (Life Technologies, Italy), according to the manufacturer on Applied Biosystems™ QuantStudio™ 5 Real-Time PCR System apparatus. Each sample was analyzed in duplicate in 20 μl of final volume containing: 6 μl Dnase/Rnase free water, 1 μl forward and reverse PCR primers at 10 μM each, and 10 μl SYBR Master Mix ready-to-use. A comparison of ATP8-6 DNA expression relative to Hexokinase 2 (HK-2) DNA expression gives a measure of mtDNA copy number to nDNA copy number ratio. The mtDNA/nDNA ratio was calculated by the 2-ΔΔCt method.

**Table 1:** Forward and reverse primers for Real-Time PCR

Primer	Sequence (5′−3′)	Gene Bank Accession number
ATP8-6 Fw	CCACAATTAAACCCAGGCCC	NC_002079.1
ATP8-6 Rev	AGGATGGGCTTGCAAATTGG	NC_002079.1
HK-2 Fw	GGCACGAATACCATCCAAGG	NC_039247.1
HK-2 Rev	CTTTCCCGTGCCGCATGAAT	NC_039247.1

### Statistics

Statistical analyses were conducted using GraphPad Prism software, version 10.0 (GraphPad Software Inc, San Diego, CA, USA). Data normality was assessed by using the Shapiro–Wilk test.

For hemodynamic analysis, comparison between curves was made by two-way ANOVA followed by Tukey post hoc test; comparison within curves was assessed by one-way ANOVA followed by Dunnett post hoc test. For all other comparisons, one-way ANOVA and Tukey’s post hoc test were applied. A *p*-value of < 0.05 was considered statistically significant. For basal hemodynamic parameters and mtDNA quantification, data on Hypo_4d group are from (Filice et al. 2024b) and were re-analyzed here in comparison to normoxia and Hypo_20d.

## Results

### Chronic hypoxia exposure improves cardiac performance

#### Cardiac basal hemodynamics

**Fig. 1:**
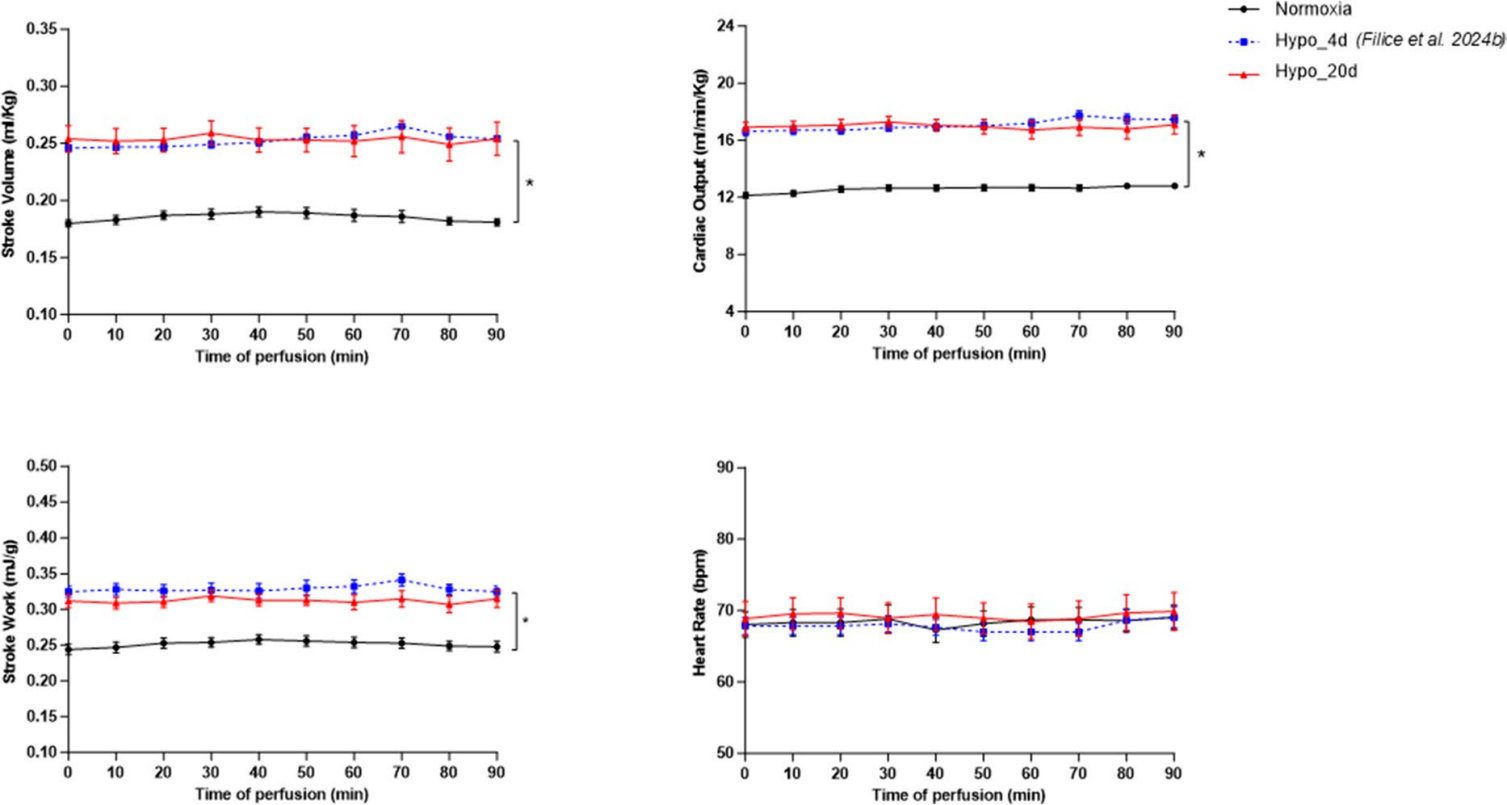
Basal hemodynamic of ex vivo cardiac preparations from normoxic- and hypoxic-exposed goldfish. Time-course curves of stroke volume, cardiac output, stroke work, and heart rate under Normoxic and Hypo_20d conditions. Curves represent the mean values ± s.e.m. (*N* = 6 for each condition). Statistics was assessed by two-way ANOVA followed by Tukey post hoc test (*p < 0.05: Hypo_20d vs. normoxia). No significant differences were observed within curves (one-way ANOVA followed by Dunnett post hoc test). For comparison, data on Hypo_4d-exposed goldfish (Filice et al. 2024b) are also reported (blue dotted lines)

Ex vivo isolated and perfused cardiac preparations from goldfish exposed to 20 days of hypoxia exhibited basal values of *Q̇*, *V*_S_, and *W*_S_ higher than those recorded in the normoxic counterpart (Table 2). As revealed by the comparison within curves, performance remained stable from the onset of perfusion and throughout the whole perfusion time (Fig. 1). No significant differences in *f*_*H*_ were recorded among groups. For comparison, the hemodynamic behavior observed in the heart from goldfish exposed to 4 days of the same level of O_2_ availability (Filice et al. 2024b) is reported (Fig. 1 blue dotted line; Table 2).

**Table 2:** Hemodynamic parameters under basal conditions

	Input pressure (kPa)	Mean output pressure (kPa)	Heart rate (bpm/min)	Stroke volume (ml Kg^−1^)	Cardiac output (ml min^−1^ kg^−1^)	Stroke work (mJ g^−1^)	Power output (mW/g)
Normoxia* (present study)*	0.071 ± 0.005	1.395 ± 0.014	68.043 ± 4.358	0.180 ± 0.008	12.140 ± 0.506	0.244 ± 0.018	0.285 ± 0.010
Hypo_4d* (*Filice et al. 2024b*)*	0.067 ± 0.001	1.390 ± 0.013	67.830 ± 2.982	0.246 ± 0.007*	16.628 ± 0.694*	0.325 ± 0.019*	0.364 ± 0.015*
Hypo_20d* (present study)*	0.072 ± 0.010	1.511 ± 0.012*^$^	68.875 ± 5.926	0.254 ± 0.028*	16.922 ± 0.849*	0.312 ± 0.023*	0.349 ± 0.008*

Basal parameters of isolated and perfused *C. auratus* cardiac preparations from animals acclimated to either normoxia (*N* = 6), or 4 days of hypoxia (Hypo_4d; < *N* = 6), or 20 days of hypoxia (Hypo_20d; *N* = 6). Statistics was assessed by one-way ANOVA followed by Bonferroni’s post hoc test (*p < 0.05: Hypo_4d or Hypo_20d vs. Normoxia; $*p* < 0.05: Hypo_20d vs. Hypo_4d). Data on Hypo_4d-exposed goldfish are from Filice et al. (2024b)

#### Frank–Starling response

**Fig. 2:**
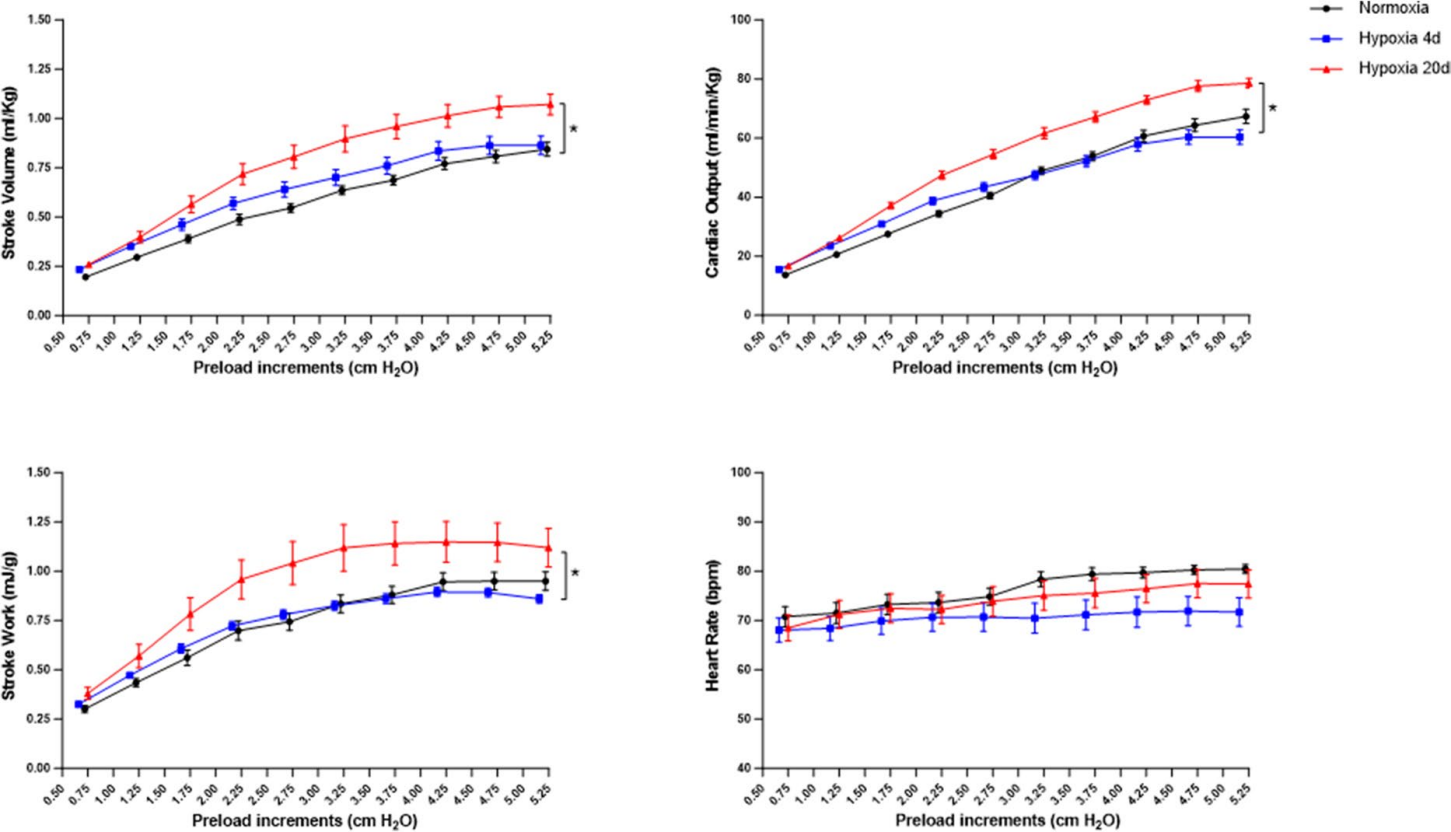
Effects of increasing preloads on cardiac hemodynamic parameters in normoxic- and hypoxic-exposed goldfish. Effects of preload increases (0.5 cmH_2_O each increment) on stroke volume, cardiac output, stroke work, and heart rate under normoxic, Hypo_4d, and Hypo_20d conditions (normoxia: *N* = 6; Hypo_4d: *N* = 5; Hypo_20d: *N* = 6). Curves represent the mean ± s.e.m. of absolute values. Statistics was assessed by two-way ANOVA followed by Tukey post hoc test (*p < 0.05: Hypo_4d vs. normoxia; $*p* < 0.05: Hypo_4d vs. Hypo_20d)

To assess whether environmental hypoxia affects the response of the goldfish heart to preload, ex vivo cardiac preparations of fish exposed to 4 and 20 days of hypoxia were evaluated under loading conditions. As shown in Fig. 2, cardiac preparations from Hypo_4d groups exhibited values of *Q̇*, *V*_*S*_, and *W*_*S*_ similar to the normoxic group. In contrast, hearts from goldfish belonging to the Hypo_20d group demonstrated a higher sensitivity to filling pressures. No differences in *f*_H_ were observed among groups (Fig. 2).

### Cardiac O_2_ consumption is time dependently modulated by hypoxia exposure

**Fig. 3:**
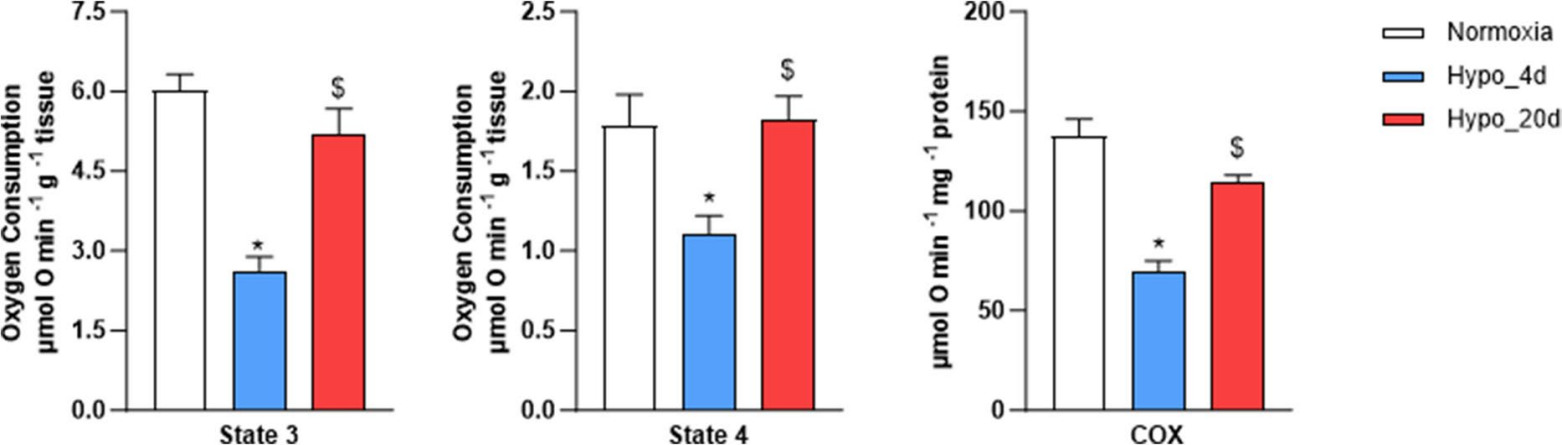
Measurements of O_2_ consumption and COX activity in the heart from normoxic- and hypoxic-exposed goldfish. O_2_ consumption measured in the presence (State 3) or absence (State 4) of ADP, and activity of the cytochrome oxidase (COX) enzyme in the cardiac muscle tissue of either Normoxia, or Hypo_4d or Hypo_20d-exposed fish (*N* = 12 for each condition). Data are expressed as means ± s.e.m. Statistics was assessed by unpaired Student’s *t*-test (*p < 0.05: Hypo_4d vs. normoxia; $*p* < 0.05: Hypo_20d vs. Hypo_4d)

O_2_ consumption during basal (State 4) and ADP-stimulated (State 3) respiration was significantly reduced after 4 days of exposure to hypoxia. Interestingly, 20 days of hypoxia restored O_2_ consumption to control levels. RCR was 3.47 ± 0.23, 2.23 ± 0.0.20, and 3.10 ± 0.18 for Normoxic, Hypo_4d, and Hypo_20d groups, respectively, with the RCR of the Hypo_4d being significantly lower than that of the other experimental groups. COX activity mirrored these changes, showing a marked decline after short-term hypoxia and a return to the control value with prolonged exposure (Fig. 3).

### Chronic hypoxia affects the expression of markers of mitochondrial biogenesis and dynamics

**Fig. 4:**
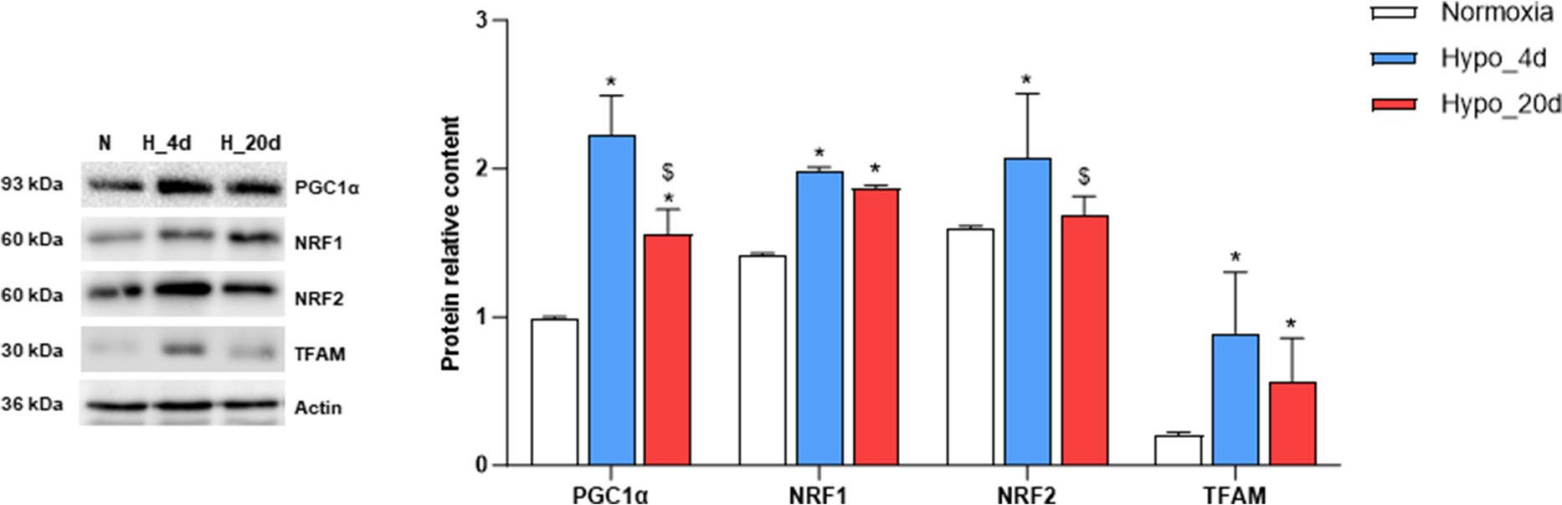
Expression of mitochondria biogenesis markers in the heart from normoxic- and hypoxic-exposed goldfish. Expression of PGC1α, NRF1, NRF2, and TFAM in cardiac extracts of goldfish exposed to either normoxia or Hypo_4d or Hypo_20d (*N* = 4 for each condition). Bar graphs display the mean ± s.e.m.; statistics was assessed by one-way ANOVA followed by Tukey post hoc test (*p < 0.05: Hypo_4d or Hypo_20d vs. normoxia; $*p* < 0.05: Hypo_4d vs. Hypo_20d)

The expression of factors regulating mitochondrial biogenesis and dynamics was assessed via Western blotting in cardiac extracts from the three experimental groups. As shown in Fig. 4, the exposure to low O_2_ affected the expression of transcription factors involved in the regulation of mitochondrial biogenesis, such as the peroxisome proliferator-activated receptor-γ coactivator (PGC)1α, the nuclear transcription factors NRF1 and NRF2, and the mitochondrial transcription factor TFAM. Compared to normoxic hearts, densitometric analyses revealed an enhanced expression of PGC1α, NRF1, and TFAM in cardiac extracts from animals exposed to both 4 and 20 days of hypoxia, with the highest increase observed at 4 days. In contrast, elevated NRF2 expression was detected only in the Hypo_4d group (Fig. 4).

**Fig. 5:**
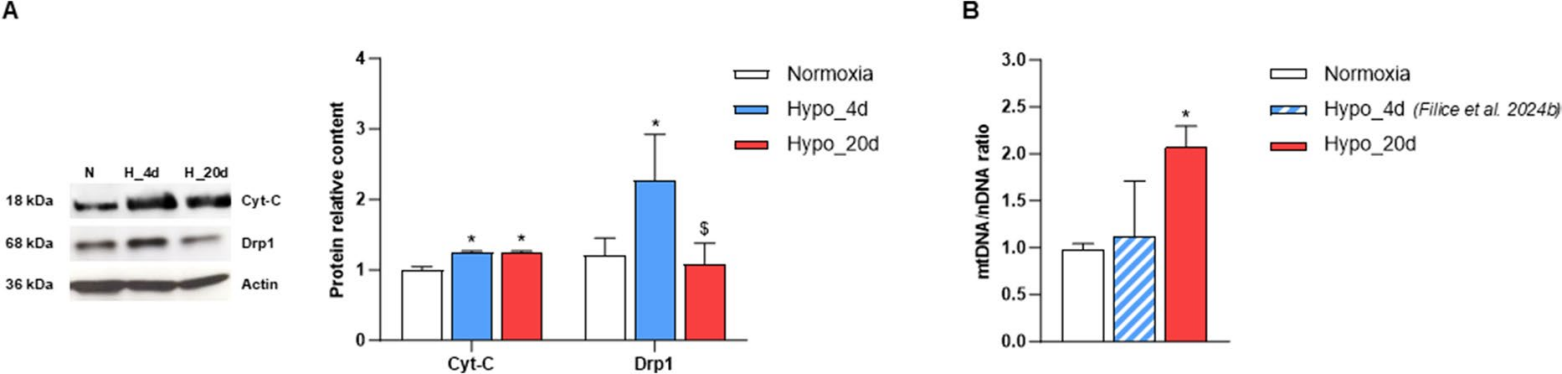
Expression of mitochondria abundance markers and mtDNA content in the heart from normoxic- and hypoxic-exposed goldfish. **A** Immunoblot and densitometric analysis of CytC and Drp1 expression in cardiac extracts of goldfish exposed to either normoxia or Hypo_4d or Hypo_20d (*N* = 4 for each condition). Bar graphs display the mean ± s.e.m.; statistics was assessed by one-way ANOVA followed by Tukey post hoc test (*p < 0.05: Hypo_4d or Hypo_20d vs. normoxia). **B** Relative mtDNA quantification on normoxic and hypoxic hearts (*N* = 4 for each condition). Statistics was assessed by one-way ANOVA followed by Tukey post hoc test (*p < 0.05: Hypo_20d vs. normoxia). For comparison, the cardiac mtDNA content of Hypo_4d-exposed goldfish (Filice et al. 2024b) is also reported (blue striped bar)

Hypoxia acclimation at both exposure times also increased the expression of Cytochrome C (Cyt-C), a biomarker of mitochondrial abundance (Napolitano et al. 2018). Otherwise, the expression of the dynamin-related protein 1 (Drp1), a marker of mitochondrial fission (Fonseca et al. 2019), only increased after short-term hypoxia (Fig. 5A).

Analysis of the cardiac mitochondrial to nuclear genome ratio (mtDNA/nDNA) indicated an increase in fish from the Hypo_20d group, with respect to the normoxia group (Fig. 5B). For comparison, data from hearts of fish exposed to 4 days of hypoxia (Filice et al. 2024b) are also included (Fig. 5B).

### Hypoxia exposure affects cardiac ROS content and induces oxidative damage to lipids

**Fig. 6:**
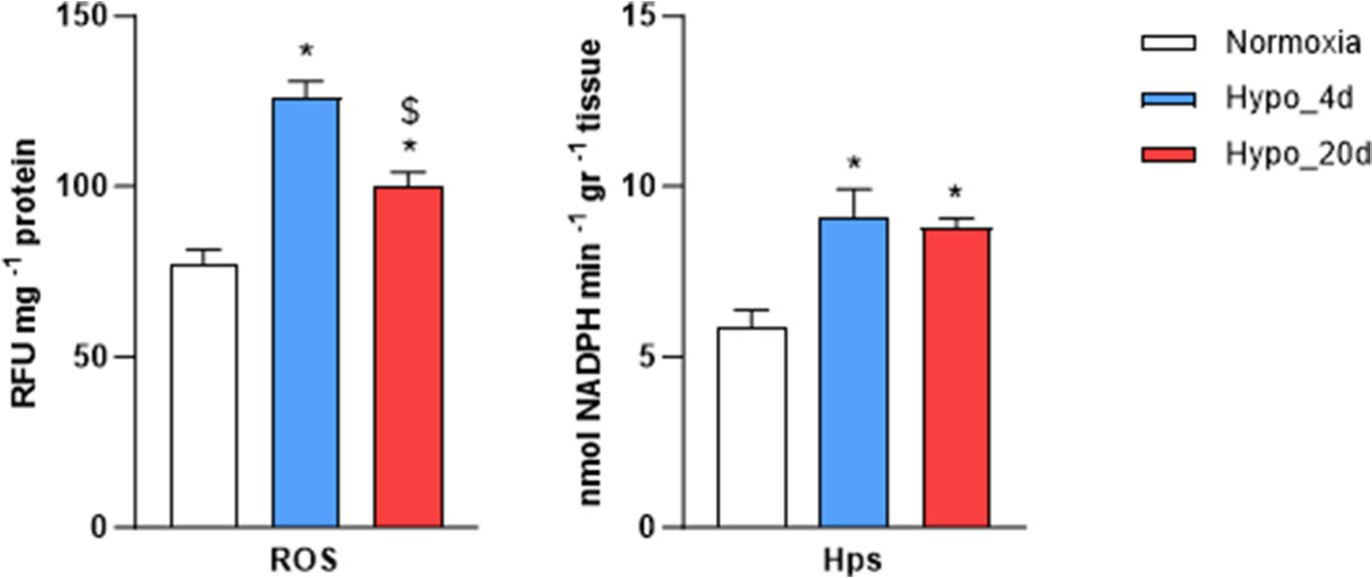
Measurement of ROS content and lipid oxidative damage in the heart from normoxic- and hypoxic-exposed goldfish. ROS content and hydroperoxides (Hps) levels in cardiac extracts from goldfish exposed to either normoxia or Hypo_4d or Hypo_20d (*N* = 12 for each condition). Data represent the mean values ± s.e.m. Statistics was assessed by one-way ANOVA followed by the Tukey post hoc test (*p < 0.05: Hypo_20d or Hypo_4d vs. Normoxia; $*p* < 0.05 Hypo_20d vs. Hypo_4d)

The contribution of reactive oxygen species (ROS) in the goldfish heart’s response to hypoxia was assessed by measuring the content of total ROS and hydroperoxides (Hps, an index of oxidative damage to lipids) in cardiac extracts from fish exposed to normoxia, as well as to 4 and 20 days of hypoxia. As illustrated in Fig. 6, hypoxia exposure led to a significant increase in the cardiac levels of total ROS and Hps, with the most pronounced response observed after 4 days of exposure.

## Discussion

This study showed that, in the goldfish, the exposure to moderate environmental hypoxia enhances the basal cardiac performance and the ability to respond to preload increases. Notably, the improved response to loading stimulation is only obtained after an extended period of exposure to low O_2_. During short-term hypoxia, these functional changes are associated with a reduced cardiac O_2_ consumption which is restored by 20 days of hypoxia. Additionally, they are accompanied by a time-dependent modulation of mitochondrial biogenesis and dynamics and myocardial oxidative status.

In teleost fish, the influence of low environmental O_2_ availability on the cardiac performance is receiving increasing attention. Studies focused on hypoxia-intolerant species show that hypoxia leads to a reduction in cardiac pumping capacity. For example, in the cod (*Gadus morhua*), both in vivo (Petersen and Gamperl 2010a) and in situ (Petersen and Gamperl 2010b) studies showed that the acclimation to a PO_2_ of 8.6 kPa for 6–12 weeks decreases hemodynamics, evaluated in terms of *V*_S_ and *Q̇*. Additionally, in the trout *Oncorhynchus mykiss* acclimated to environmental moderate hypoxia (> 6 weeks at 8.5 kPa), an impaired myocardial performance is reported (Motyka et al. 2017). This is mainly attributed to a reduced ability of the heart to eject blood, as shown by a decreased ejection fraction (Carnevale et al. 2020).

A different cardiac behavior is observed in species that are able to tolerate even dramatic O_2_ deprivation, as in the case of cyprinids. An example is the crucian carp exposed to anoxia in which, after an initial increase of *V*_S_, *Q̇*, *f*_H_, and power output, cardiac hemodynamic parameters return to the pre-anoxic values within 24 h, to remain stable up to 5 days of anoxia (Stecyk et al. 2004). In line with these data, we recently documented that heart preparations from goldfish exposed to 4 days of environmental moderate hypoxia reach values of *V*_S_ and *Q̇* higher than those detected in the normoxic counterpart (Filice et al. 2024b). Interestingly, in the present study, we observed that this cardiac behavior is maintained also when goldfish are exposed for 20 days to similar levels of O_2_ deprivation. In fact, hemodynamic analyses carried out on the ex vivo isolated and perfused goldfish heart revealed that the basal values of *V*_S_, *Q̇*, and *W*_S_ are higher than those detected under normoxia but are comparable to those recorded in 4 days hypoxia-exposed fish (Table 1). Interestingly, a time-dependent increase of the hemodynamic performance has been already reported also in the heart from animals acclimated to water normoxia and acutely perfused with low O_2_ (Imbrogno et al. 2014). Taken together, these observations suggest that in the presence of hypoxia, the goldfish heart can rapidly enhance its basal performance within approximately 2 h (Imbrogno et al. 2014), thus achieving high values of *V*_S_, *Q̇*, and *W*_S_. The enhanced cardiac performance is maintained even with prolonged exposure to hypoxic stress (up to 20 days). This likely enables the fish to sustain high blood flow, ensuring adequate organ and tissue perfusion while facilitating lactate transport to muscle for conversion into ethanol (Cerra et al. 2023; Imbrogno et al. 2019, 2022).

When the cardiac performance of goldfish acclimated to 4 and 20 days of hypoxia was analyzed under increasing hemodynamic loads, different shapes of the Frank–Starling curve were obtained (Fig. 2). Cardiac preparations from fish exposed to 4 days of hypoxia showed a sensitivity to filling pressure changes similar to the normoxic counterpart. Otherwise, the heart from fish of the Hypo_20d group exhibited an improved sensitivity to preload increments, being able to reach values of *V*_S_, *Q̇*, and *W*_S_ higher than those recorded in both normoxic and Hypo_4d groups. The Frank–Starling mechanism is a major regulator of myocardial contractility in all vertebrates (Shiels and White 2008). It allows the myocardium to respond to filling pressure increases with a more vigorous contraction, enabling the heart to perform additional work to augment *V*_S_, and thus *Q̇*. The fish heart is particularly sensitive to preload increases [see for example (Amelio et al. 2013; Cerra et al. 2004; Filice et al. 2021; Garofalo et al. 2009; Gattuso et al. 2002; Icardo et al. 2005; Imbrogno et al. 2013; Olson 1998)], a feature that is correlated with the great extensibility of the trabeculated heart, associated to a high calcium sensitivity at a large range of sarcomere lengths (Di Maio and Block 2008; Shiels et al. 2006; Shiels and White 2008). A marked response to filling pressure also characterizes the hemodynamics of the goldfish heart. This is evidenced by the elevated *V*_S_ values expressed at increasing preloads under normoxia (Garofalo et al. 2012) and by the even higher sensitivity observed under acute hypoxia (Imbrogno et al. 2014). Together with these data, the results here obtained on the heart from the Hypo_20d group strongly support the possibility that the hypoxic challenge improves the goldfish cardiac performance not only under basal conditions but also in response to preload stimulations. This reinforces the key role of the Frank–Starling response in adapting the cardiac performance of teleost fish to challenging hemodynamic requests.

The relationship between increased cardiac mechanical performance and heightened ATP demand is well established (Gibbs and Loiselle 2001). Cardiac bioenergetics relies heavily on the coordination of metabolic processes that regulate mitochondrial respiration and energy flow within cellular compartments, thereby ensuring ATP availability for myocardial contraction (Saks et al. 2006). A limited supply of O_2_ can disrupt this delicate balance, impairing cardiac bioenergetics, as observed in hypoxia-sensitive mammalian myocardium (Hernandez-Resendiz et al. 2023).

In the present study, we observed that the exposure of goldfish to water hypoxia elicited time-dependent changes in ventricular mitochondrial respiration. This was revealed by a reduced O_2_ consumption observed under both basal (State 4) and ADP-stimulated (State 3) conditions in the Hypo_4d group and by the return to normoxic values in the Hypo_20d group. Compared to control goldfish, O_2_ consumption in the Hypo_4d group was reduced by 36% in State 4 and 56% in State 3. Thus, the basal respiration, which depends on proton leak and electron chain activity, is less affected by hypoxia than ADP-stimulated respiration. Consequently, a significant decrease of RCR was also observed in goldfish exposed to hypoxia for four days. Since RCR is an index of the coupling between respiration and ADP phosphorylation, this suggests that the ATP synthesis is reduced under short-term exposure to hypoxia. A similar time-dependent pattern was observed in COX activity, with a reduction in the Hypo_4d group and a return to normoxia-like values in the Hypo_20d group. The behavior of fish exposed for long-term to hypoxia agrees with evidence from Farhat and colleagues showing that goldfish exposed to severe hypoxia for 4 weeks maintain a normal cardiac mitochondrial respiratory capacity and COX activity (Farhat et al. 2021). A downregulation of the respiratory capacity is a strategy adopted by several anoxia-tolerant animals to preserve energy during chronic O_2_ deprivation (Galli and Richards 2014). It also contributes in preventing the ATP synthase from running in reverse mode, a mechanism commonly activated under hypoxia in the attempt to maintain inner membrane potential and cellular energy status (Galli and Richards 2014). Together with this information, our data suggest that, when goldfish is exposed for a short time to low water O_2_, the heart undergoes a transient reduction in mitochondrial respiration, which may contribute to stabilize the mitochondrial membrane potential, thus allowing to rapidly cope with hypoxic stress. Over longer exposure, mitochondrial function is restored, and this may represent an adaptive response to sustain myocardial energy demands if hypoxia persists, as occurring in the natural environments.

**Fig. 7:**
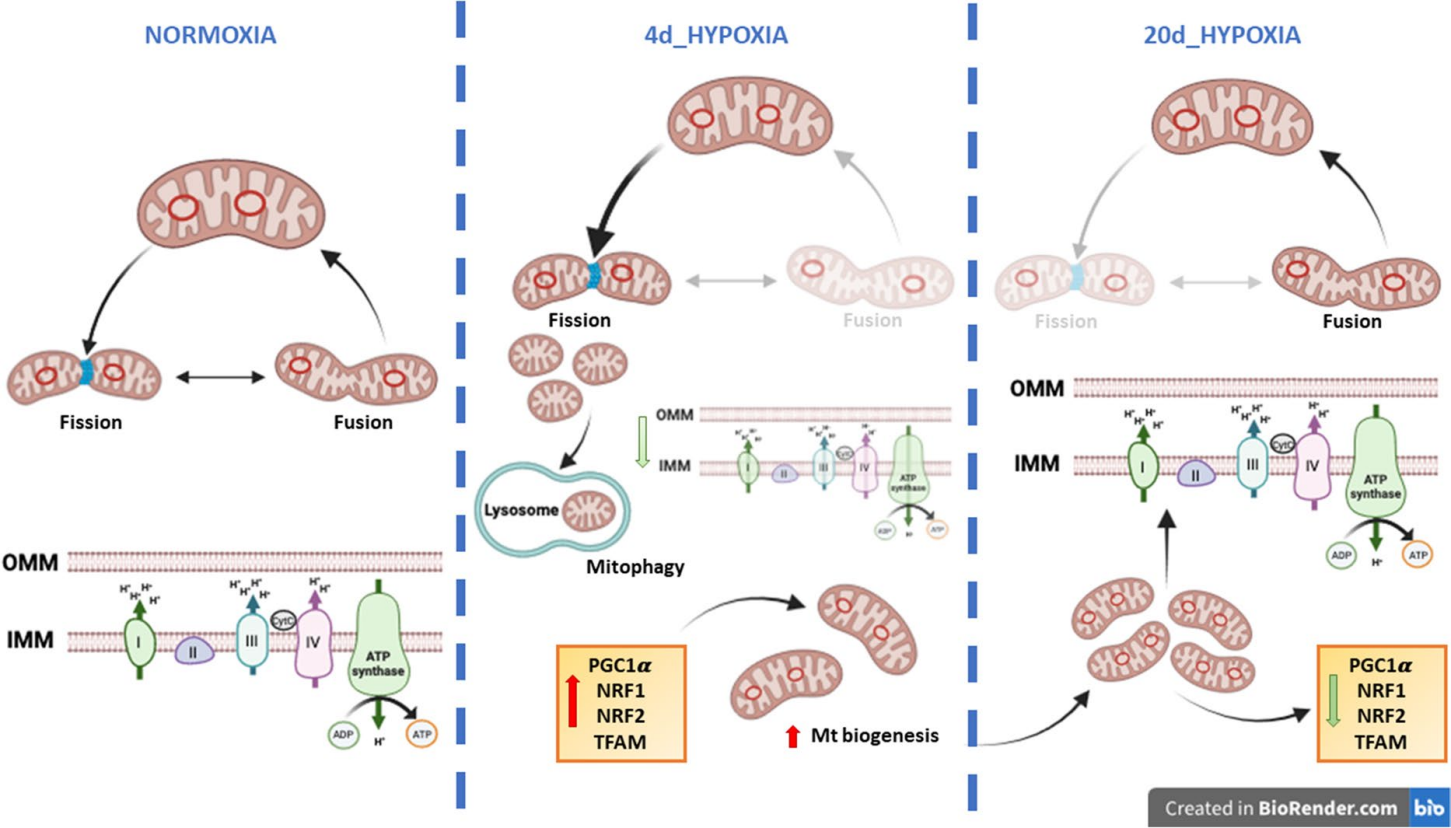
Simplified diagram of the mechanisms involved in the mitochondrial response to environmental moderate hypoxia in the goldfish heart. Under normoxia, the maintenance of mitochondrial integrity and homeostasis is achieved through a balance between fusion and fission events. In the short term of hypoxia exposure, the decrease in mitochondrial respiration is accompanied by an enhanced fission and by initial signs of biogenesis. The rapid de novo production may replenish the mitochondrial pool, weakened by fission. Under protracted hypoxia, mitochondrial respiration is recovered, fission decreases, and biogenesis is reduced. The reinforcement of the mitochondrial compartment may allow to supply the myocardium with adequate energy, enabling it to sustain the enhanced costs of the response to increased loadings

Interestingly, in the goldfish heart, also mitochondrial dynamics appears to be sensitive to the duration of hypoxia exposure. We previously demonstrated that 4 days of hypoxia are associated with an increase in the mitochondrial compartment, together with the activation of fission events. This is documented by the enhanced expression of pro-fission proteins, including Drp1 (Filice et al. 2024b). In contrast, we here demonstrated that, when the exposure to water hypoxia is protracted up to 20 days, the cardiac expression of Drp1 decreases to the levels found under normoxia. This is consistent with data from Farhat and colleagues (Farhat et al. 2022), which reported an increase in pro-fusion transcripts in goldfish hearts after 4 weeks of hypoxia. Fission and fusion events play a key role in determining mitochondrial shape, and the balance between these two opposite processes maintains organelle number, mtDNA mixing, and function (Adebayo et al. 2021). In mammals, decreased mitochondrial respiration is associated with increased fission (Parra et al. 2017), which facilitates mitophagy to remove damaged mitochondria (Galloway and Yoon 2015; Napolitano et al. 2023). This is a characteristic of several myocardial diseases, including those linked to reduced O_2_ supply, in which an excessive fission of mitochondria is detrimental for the heart (Hernandez-Resendiz et al. 2023). Different from mammals, in the goldfish exposed to 4 days of hypoxia, the enhanced mitochondrial fission and the reduced respiration are not detrimental for the heart that, under these conditions, enhances its pumping performance (Filice et al. 2024b). Thus, the possibility exists that, in the goldfish, other events may sustain the myocardial energy demand. We here found that, the exposure to hypoxia increases the cardiac expression of the transcriptional co-activator PGC1α, of the nuclear transcription factors 1 and 2 NRF1, NRF2, and of the mitochondrial DNA transcription factor TFAM. This is particularly evident in the heart of Hypo_4d animals and persisted, albeit at lower levels, in the Hypo_20d group. An increased expression of the above factors is indicative of mitochondrial biogenesis and correlates with changes in mitochondrial content (Venditti and Di Meo 2020). We hypothesized that in the goldfish, a rapid activation of mitochondrial biogenesis during short-term hypoxia exposure may help replenish the mitochondrial pool, possibly weakened by fission events (Filice et al. 2024b). If hypoxia persists, mitochondrial biogenesis may decrease as the mitochondrial compartment stabilizes (Fig. 7). This possibility is supported by our results showing a reduced expression of PGC1α, NRF1, NRF2, and TFAM in the heart of the Hypo_20d group, in parallel with an enhancement of cytC expression and mtDNA/nDNA ratio. This pattern differs from the behavior observed in the skeletal muscle of goldfish exposed to short and/or prolonged hypoxia where PGC1α and NRF2 expression is modulated without corresponding changes in TFAM or markers of mitochondrial abundance (Filice et al. 2024a) and function (Farhat et al. 2021; Thoral et al. 2022). These results suggest that hypoxia exposure may trigger tissue-specific modulation of the mitochondrial apparatus, reflecting the distinct energy demands of different tissues.

Of note, the mitochondrial remodeling observed in the goldfish heart under hypoxia is paralleled by changes in the oxidative status. In fact, we observed that both total ROS content and lipid peroxidation increased under short hypoxia, to decrease when hypoxia is protracted up to 20 days. In the mammalian heart, when O_2_ availability is limited, mitochondria are impaired, and this correlates with a higher ROS production (Hernandez-Resendiz et al. 2023). Differently, in hypoxia tolerant fish, exposure to low O_2_ is accompanied by either unchanged [*Anoplopoma fimbria*; (Gerber et al. 2019)] or even decreased [*Fundulus heteroclitus*; (Du et al. 2016)] ROS release. Further investigation is needed to clarify the role of oxidative changes in the goldfish heart during different temporal phases of the hypoxic response and to assess their potential correlation with mitochondrial efficiency.

## Conclusions

The results from the present study, together with our recent findings (Filice et al. 2024a, b), provide information on the temporal framework of hemodynamic and mitochondrial events that characterize the adaptation of the goldfish heart to moderate hypoxia. The general response is an improvement of the cardiac performance which, only after prolonged exposure, is characterized by a high sensitivity to loading stimulations. This contrasts with the hypoxia-sensitive mammalian myocardium, whose performance is impaired in the presence of hypoxia (Hernandez-Resendiz et al. 2023). In the short term, the enhanced hemodynamics of the goldfish heart was associated with a decrease in mitochondrial respiration, accompanied by increased mitochondrial fission and early signs of biogenesis. The rapid de novo production may start to replenish the mitochondrial pool, which is initially weakened by fission. With extended hypoxia, fission was no more present, mitochondrial respiration was recovered, and biogenesis was reduced. The reinforcement of the mitochondrial compartment may allow to supply the myocardium with adequate energy, enabling it to sustain also the enhanced costs of the response to increased loadings (Fig. 7).

Overall, the data provided by this study emphasize the importance of further investigating the goldfish heart to uncover the molecular mechanisms that allow it to maintain and even potentiate its performance in the presence of the constraints imposed by reduced O_2_ availability. This is a challenging task with putative implications not only for basic physiological research but also under a wider perspective that spans from environmental/conservation to translational applications.

## Data Availability

No datasets were generated or analysed during the current study.

## Abbreviations

COX: Cytochrome c oxidase
Drp1: Dynamin-related protein 1
*f*_H_: Heart rate
NRF1: Nuclear transcription factor 1
NRF2: Nuclear transcription factor 2
PGC1α: Peroxisome proliferator-activated receptor-γ coactivator
Pi: Filling pressure
*Q̇*: Cardiac output
ROS: Reactive oxygen species
TFAM: Mitochondrial transcription factor
*W*_S_: Stroke work
*V*_S_: Stroke volume
